# Diffuse large B-cell non-Hodgkin lymphomas: the clinical relevance of histological subclassification

**DOI:** 10.1038/sj.bjc.6690282

**Published:** 1999-04

**Authors:** J W Baars, D de Jong, E M Willemse, L Gras, O Dalesio, P v Heerde, P C Huygens, H v d Lelie, A E G Kr v.d. Borne

**Affiliations:** Division of Medical Oncology, Department of Hematology, Department of Pathology, Department of Scientific and Patient Administration, Department of Biometrics, Netherlands Cancer Institute/Antoni van Leeuwenhoek Hospital, Plesmanlaan 121, Amsterdam, 1066 CX, The Netherlands; Department of Hematology, Academic Hospital of the Free University, de Boelelaan 1117, Amsterdam, 1081 HV, The Netherlands; Division of Internal Medicine, Department of Hematology, Academic Medical Center, Meibergdreef 9, Amsterdam, 1105 AZ, The Netherlands

**Keywords:** diffuse large B-cell NHL, diffuse centroblastic NHL, immunoblastic NHL, centroblastic polymorphic subtype NHL, subclassification, prognostic significance

## Abstract

In the REAL classification the diffuse large B-cell non-Hodgkin lymphomas (NHL) are grouped together, because subclassifications are considered to lack both reproducibility and clinical significance. Others, however, claim that patients with an immunoblastic NHL have a worse prognosis than patients with other types of diffuse large B-cell NHL. Therefore, we investigated the prognostic and clinical significance of histological subclassification of diffuse large B-cell NHL in a uniformly treated series of patients. For this retrospective study, all patients diagnosed as having an immunoblastic (IB) B-cell NHL by the Lymphoma Review Panel of the Comprehensive Cancer Center Amsterdam (CCCA) between 1984 and 1994, and treated according to the guidelines of the CCCA, were analysed. Patients with a centroblastic polymorphic subtype (CB-Poly) or centroblastic (CB) NHL by the Lymphoma Review Panel who were treated in the Netherlands Cancer Institute during the same period according to CCCA guidelines were used as reference groups. All patients' records were reviewed. Clinical parameters at presentation, kind of therapy and clinical outcome were recorded. All available histological slides were separately reviewed by two haemato-pathologists. One hundred and seventy-seven patients were included in the study: 36 patients (20.3%) with an IB NHL, 69 patients (39%) with a CB-Poly NHL and 72 patients (40.7%) with a CB NHL. The patients with an IB NHL tended to be older and presented more often with stage I or II and one extranodal site than patients with a CB and CB-Poly NHL. None of the subtypes showed a clear preference for localization in a particular site. The patients with IB or CB-Poly NHL showed a significantly worse prognosis than patients with CB NHL, with a 5-year overall survival for patients with CB NHL of 56.3% and for patients with IB or CB-Poly NHL 39.1% and 41.6% respectively. The 5-year disease free survival was 53.2% for the patients with CB, 32% for the patients with CB-Poly and 26.9% for the patients with IB NHL. A multivariate analysis showed that histological subtyping was of prognostic significance independent of the International Prognostic Index. This finding merits further exploration in prospective studies in order to judge the value of subclassification of large B-cell NHL as a guideline in therapy choice. © 1999 Cancer Research Campaign


*British Journal of Cancer* (1999) **79**(11/12), 1770–1776
© 1999 Cancer Research Campaign
Article no. bjoc.1998.0282

### Diffuse large B-cell non-Hodgkin lymphomas: the clinical relevance of histological subclassification

**JW Baars[1], D de Jong[2], EM Willemse[3], L Gras[4], O Dalesio[4], P v. Heerde[2], PC Huygens[5], H v.d. Lelie[6] and AEG Kr v.d. Borne[1,6]**

[1]Division of Medical Oncology, Department of Hematology, [2]Department of Pathology, [3]Department of Scientific and Patient Administration, [4]Department of Biometrics, Netherlands Cancer Institute/Antoni van Leeuwenhoek Hospital, Plesmanlaan 121,1066 CX Amsterdam, The Netherlands; [5]Department of Hematology, Academic Hospital of the Free University, de Boelelaan 1117, 1081 HV Amsterdam, The Netherlands; [6]Division of Internal Medicine, Department of Hematology, Academic Medical Center, Meibergdreef 9, 1105 AZ Amsterdam, The Netherlands

**Summary** In the REAL classification the diffuse large B-cell non-Hodgkin lymphomas (NHL) are grouped together, because subclassifications are considered to lack both reproducibility and clinical significance. Others, however, claim that patients with an immunoblastic NHL have a worse prognosis than patients with other types of diffuse large B-cell NHL. Therefore, we investigated the prognostic and clinical significance of histological subclassification of diffuse large B-cell NHL in a uniformly treated series of patients. For this retrospective study, all patients diagnosed as having an immunoblastic (IB) B-cell NHL by the Lymphoma Review Panel of the Comprehensive Cancer Center Amsterdam (CCCA) between 1984 and 1994, and treated according to the guidelines of the CCCA, were analysed. Patients with a centroblastic polymorphic subtype (CB-Poly) or centroblastic (CB) NHL by the Lymphoma Review Panel who were treated in the Netherlands Cancer Institute during the same period according to CCCA guidelines were used as reference groups. All patients' records were reviewed. Clinical parameters at presentation, kind of therapy and clinical outcome were recorded. All available histological slides were separately reviewed by two haemato-pathologists. One hundred and seventy-seven patients were included in the study: 36 patients (20.3%) with an IB NHL, 69 patients (39%) with a CB-Poly NHL and 72 patients (40.7%) with a CB NHL. The patients with an IB NHL tended to be older and presented more often with stage I or II and one extranodal site than patients with a CB and CB-Poly NHL. None of the subtypes showed a clear preference for localization in a particular site. The patients with IB or CB-Poly NHL showed a significantly worse prognosis than patients with CB NHL, with a 5-year overall survival for patients with CB NHL of 56.3% and for patients with IB or CB-Poly NHL 39.1% and 41.6% respectively. The 5-year disease free survival was 53.2% for the patients with CB, 32% for the patients with CB-Poly and 26.9% for the patients with IB NHL. A multivariate analysis showed that histological subtyping was of prognostic significance independent of the International Prognostic Index. This finding merits further exploration in prospective studies in order to judge the value of subclassification of large B-cell NHL as a guideline in therapy choice.

**Keywords:** diffuse large B-cell NHL; diffuse centroblastic NHL; immunoblastic NHL; centroblastic polymorphic subtype NHL; subclassification, prognostic significance

The diffuse large B-cell non-Hodgkin lymphomas (NHL) encompass a heterogeneous group of NHL with respect to the morphological spectrum and clinical behaviour. In the Kiel classification, diffuse large B-cell NHL are subclassified on the basis of their morphology in three main categories of diffuse centroblastic lymphoma (including the monomorphic, polymorphic, centrocytoid and multilobated subtypes), B-immunoblastic lymphoma and anaplastic large cell lymphoma (Lennert and Feller, 1990). Patients with an immunoblastic NHL were always considered to have a significantly worse prognosis (Rosenberg et al, 1982; Stein and Dallenbach, 1992). On the basis of the analysis of the original series of the Working Formulation (WF), immunoblastic NHL was set apart from the other large B-cell NHL and considered to be of high-grade malignancy (Rosenberg et al, 1982; Stein and Dallenbach, 1992; van Heerde et al, 1996). These views have been challenged, however, by other investigators. In the proposed Revised European–American Classification of Lymphoid Neoplasms (REAL classification), the diffuse large B-cell lymphomas are grouped together, because of a presumed lack of reproducibility of histologic subclassification in daily practice and, therefore, subtyping of the diffuse large B-cell NHL is considered to be of minor clinical significance (Harris et al, 1994; Berard and Hutchison, 1997).

Preliminary presentations of the new WHO classification show that the view of the REAL classification is largely adopted (Jaffe et al, 1997). Specific morphological variants have been defined with the purpose of recognizing diagnostic pitfalls, including, for example, anaplastic, T-cell/histiocyte rich, centroblastic and immunoblastic NHL. A clinical significance of these subtypes is, however, not implicated. Only primary mediastinal (thymic) large B-cell NHL and intravascular large B-cell NHL are listed as separate clinico-pathological entities on the basis of their specific presentation and clinical course (Jaffe et al, 1997).

In this retrospective study, we analysed whether subclassification of large B-cell NHL, concentrating on the original Kiel classification criteria of B-immunoblastic, centroblastic polymorphic

*Received 24 April 1998*
*Revised 24 July 1998*
*Accepted 13 October 1998*

*Correspondence to:* Mrs Dr JW Baars subtype and centroblastic (including monomorphic, centrocytoid and multilobated subtypes) NHL, is of clinical significance with regard to parameters at presentation, including specific localizations, response to therapy, patterns of relapse as well as clinical outcome (overall and disease-free survival), in order to investigate whether it may be justifiable to draw therapeutic consequences from the histological subclassification of large B-cell NHL.

### PATIENTS AND METHODS

### Patients

For this retrospective study, all patients diagnosed as having an immunoblastic B-cell NHL were retrieved from the files of the Lymphoma Review Panel of the Comprehensive Cancer Center Amsterdam (CCCA) ($n$ = 45). Patients treated according to the guidelines of the CCCA between 1984 and 1994 were analysed for this study. Patients diagnosed as having a centroblastic NHL (monomorphic, centrocytoid and multilobated subtypes, further called centroblastic NHL) and centroblastic polymorphic subtype NHL by the CCCA Lymphoma Review Panel and who were treated in the same period according to the CCCA guidelines in the Netherlands Cancer Institute were used as reference groups ($n$ = 198). The patients included into this study were all previously untreated. The patients with a centroblastic or centroblastic polymorphic NHL were all treated in the Netherlands Cancer Institute. From the 45 patients with an immunoblastic NHL, 30 patients were treated in the Netherlands Cancer Institute and 15 patients in 5 other hospitals connected to the Comprehensive Cancer Center Amsterdam. As stated above, the treatment guidelines were similar for all institutes.

Clinical records were reviewed (JWB, EMW) and the following parameters at presentation were recorded: age, stage, performance status, sex, LDH, number and localization of extranodal sites, bulky disease (defined as a mass ≥ 5 cm) and the presence of B-symptoms. The therapeutic regimen was recorded, including information on the composition of the chemotherapeutic schedules, dose reduction of the chemotherapy and radiotherapy data. Response to therapy, time to relapse, second-line therapy for relapses, time and cause of death, date last seen and disease status at the time of review (alive with or without disease) were included.

Patients were excluded from this study on the following criteria: a recognized disease phase of a follicular centrocytic/centroblastic NHL or another type of low-grade B-cell NHL, with subsequent transformation to a large B-cell NHL, human immunodeficiency virus (HIV) related NHL, secondary NHL after previous treatment of an unrelated malignancy and proven T-cell phenotype upon review. Patients of whom no histological slides were available for review and patients with inadequate histological or incomplete clinical data (two or more prognostic parameters lacking) were also excluded from this study.

### Histology

All cases were originally diagnosed and classified according to the Kiel-classification and Working Formulation by the CCCA Lymphoma Review Panel, consisting of at least three experienced haematopathologists. In all cases, a minimum panel of immunohistochemical markers was available, including CD20 (L26), MB2, CD45RO (UCHL 1), CD43 (MT1) and CD45RA (LCA) and in later years also including CD3, CD30 and CD79a (JCB 117).

From 229 cases (45 patients with immunoblastic NHL and 184 patients with centroblastic or centroblastic polymorphic subtype NHL), histological slides were still available for review, and these cases were reclassified according to the updated Kiel classification by two haematopathologists, independently from each other (DdJ, PvH). In case of discrepancy between the reviewers or with the original CCCA panel diagnosis, cases were reviewed together to come to a consensus diagnosis. Immunohistochemical studies were completed, if necessary. According to the criteria defined by the updated Kiel classification (Lennert and Feller, 1990) (and adopted by the recent WHO classification proposal), immunoblastic B-NHL was defined by a tumour cell population of ≥ 90% immunoblasts, centroblastic NHL as ≤ 10% immunoblasts and centroblastic polymorphic subtype NHL as between 10–90% immunoblasts. A further subdivision was recorded as group 1 = 10–25% immunoblasts, group 2 = 25–50% immunoblasts, group 3 = 50–75% immunoblasts and group 4 = 75–90% immunoblasts of the total malignant B-cell infiltrate. In order to investigate the additional value of the immunohistochemical identification of immunoblasts in quantifying their number, an immunohisto chemical analysis was performed with the plasma cell-related antibodies CD138 (Syndecan-1, 1D4/B-B4 antibody, Serotec Ltd, Oxford, UK) (Wijdenes et al, 1996) and VS38c (Dako A/S, Glostrup, Denmark) on 19 cases of immunoblastic NHL, 21 cases of centroblastic NHL and 21 cases of polymorphic centroblastic NHL. Subclassification on the basis of this additional immunohistochemical information was performed without knowledge of the previous diagnosis.

### Statistical analysis

Overall survival and disease-free survival curves were estimated using the Kaplan–Meier method. The univariate associations between different clinical and histological features with overall and disease-free survival were tested with the log-rank test. Stratified log-rank tests were performed to study the prognostic value of histological subclassification, adjusting for the international prognostic index (Shipp et al, 1993). In addition to the stratified log-rank tests, the Cox proportional hazards model was used. A step forward procedure was performed to study the risk associated with different histological subtypes, adjusting for the international prognostic index (Shipp et al, 1993), sex, B-symptoms, bulky disease (≥ 5 cm) and bone marrow involvement. Association of different clinical features with histological cell type was studied using the Pearson chi-square test. All reported $P$-values were obtained from two-sided tests. $P$-values of > 0.05 are reported as not significant.

### RESULTS

### Histology

Forty-five cases with immunoblastic NHL and 198 cases with centroblastic or centroblastic polymorphic subtype were retrieved from the files of the Lymphoma Review Panel of the CCCA between 1984 and 1994.

After exclusion according to the above-mentioned criteria, 177 patients with large B-cell NHL were included in this study. Thirty-six (20.3%) of the patients were diagnosed to have an immunoblastic B-cell NHL (IB NHL). Of the 45 originally retrieved cases of immunoblastic NHL, 2 cases were excluded bacause of lack of enough clinical data, 3 cases were excluded on the basis of not-proven B-cell phenotype (most probably T-NHL), 3 cases were reclassified as centroblastic polymorphic subtype NHL upon review (group 4, between 75 and 90% immunoblasts) and 1 patient was recognized as having a follicular centrocytic/centroblastic NHL transformed to an immunoblastic B-cell NHL at the time of the first presentation.

Of the 198 originally retrieved cases of centroblastic NHL or centroblastic polymorphic subtype NHL, 57 cases were excluded for the following reasons: no histological slides available for review (14 cases), inadequate histology (3 cases), incomplete clinical data (27 cases), HIV-related NHL (9 cases), follicular centroblastic/centrocytic NHL transformed to a centroblastic NHL and centroblastic polymorphic subtype respectively at the time of presentation (2 cases) and development of a large B-cell NHL after previous treatment for Hodgkin's disease (2 cases). Of the 141 included cases, 69 (39%) of the cases were classified as centroblastic polymorphic subtype NHL (CB-Poly NHL) and 72 (40.3%) as centroblastic NHL (CB NHL). In 8/141 (5.7%) cases, review resulted in divergence of classification between the centroblastic and centroblastic polymorphic subtype (2 patients originally diagnosed to have a CB NHL had a CB-Poly NHL upon review, 6 patients originally diagnosed to have a CB-Poly NHL had a CB NHL upon review). No cases with a centroblastic or centroblastic polymorphic subtype NHL were reclassified as immunoblastic NHL. Thus, in the total group of 177 patients, divergence in histological subclassification between the two haematopathologists occurred in 11 patients (6.2%). The intra-observer variability for the two haematopathologists (all slides were reviewed twice with an interval of at least 2 months) were 6% ($n$ = 10) and 10% ($n$ = 18) respectively. In case of discrepancy between the first and second observation and in case of discrepancy between the two observers, a consensus diagnosis was made between the two haematopathologists.

Immunohistochemical staining with CD138 (Syndecan-1, 1D4/B-B4) and VS38c in 61 cases of large B-cell NHL showed strong staining of reactive plasma cells in all cases. Both markers were quite sensitive to decline of staining intensity upon long-term storage of archival unstained paraffin sections. With optimal tissue quality, reliable and reproducible staining of immunoblasts could be achieved with a lower intensity than the concomitant reactive plasma cells (Figure 1). Overall, immunohistochemical identification of immunoblasts with CD138 and VS38c was insufficiently reliable to add significantly to the quantification of immunoblasts and is probably not useful for the subclassification of large B-cell NHL.

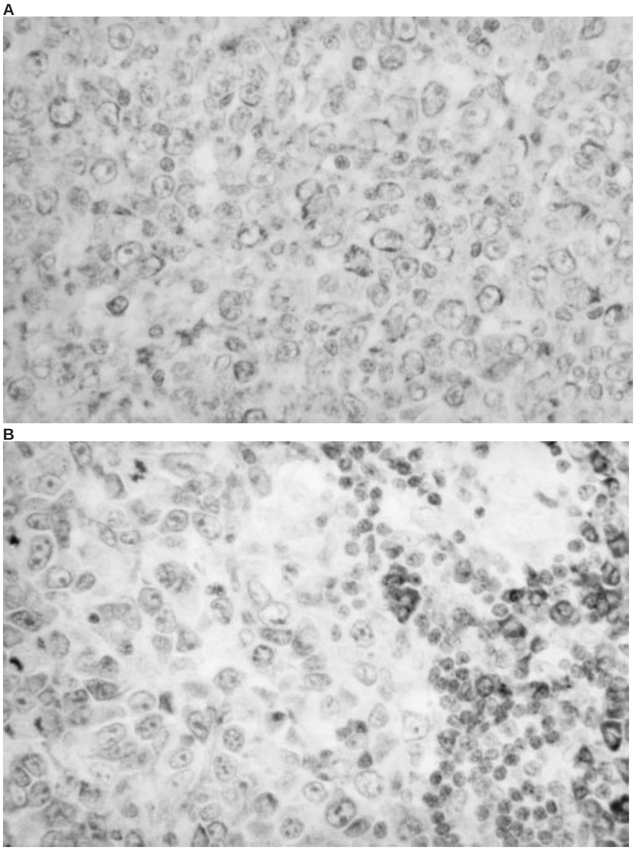

**Figure 1** Immunohistochemical staining with CD138 (Syndecan-1, 1D4/B-B4) (**A**) and VS38C (**B**) in 2 cases of immunoblastic NHL. Immunoblasts show a lower staining intensity than concomitant plasma cells

### Clinical parameters at presentation

The clinical characteristics at presentation are summarized in Table 1.

Patients with an immunoblastic NHL tended to be older than patients with polymorphic centroblastic or centroblastic NHL. The patients with an immunoblastic NHL presented more frequently with stage I or II and one extranodal site than patients with a polymorphic centroblastic or centroblastic NHL. No major differences were found between the subentities of the diffuse large B-cell NHL with regard to the other prognostic criteria according to the International Prognostic Index (IPI) according to Shipp et al (1993: performance status, serum concentrations of LDH, number of extranodal sites [≤ 1 versus > 1], stage). In addition, the distribution of prognostic groups according to the IPI (Shipp et al, 1993) was similar for the three histologic subentities (Table 1).

The patients with polymorphic centroblastic NHL tended to present more often with bone marrow involvement than patients with immunoblastic or centroblastic NHL (Table 1).

The extranodal NHL localizations at presentation are summarized in Table 2. It should be noted that none of the subtypes of large B-cell NHL showed preference for localization in a particular site, except localization of centroblastic NHL in the thyroid. The numbers are, however, too small to draw definite conclusions.

There were no differences in localization and frequency of extranodal relapses.

### Therapy

The majority of the patients (52%, 92/177) were treated with CHOP-(like) regimens and radiotherapy. Thirty-four patients (19.2%) received only CHOP-like regimens, 47 patients (26.6%) were treated only with radiotherapy (the majority of the patients treated only with radiotherapy had stage I or II and were treated before 1990). According to the CCCA guidelines, 11 of these 47 patients should have received chemotherapy because of stage and/or bulky disease. (Anthracycline containing) chemotherapy was omitted for the following reasons: erroneous initial diagnosis of seminoma in a patient with centroblastic polymorphic subtype NHL, patient's refusal (1 patient with centroblastic NHL, 1 patient

 © Cancer Research Campaign 1999

**Table 1** Clinical parameters at presentation of 177 patients with large B-cell NHL

| | IB NHL[a] | CB-Poly NHL | CB NHL | *P*-value |
|---|---|---|---|---|
| No. of patients | 36 | 69 | 72 | |
| Sex | | | | |
| M | 19 (52.8%) | 34 (49.3%) | 35 (48.6%) | |
| F | 17 (47.2%) | 35 (50.7%) | 37 (51.4%) | 0.916 |
| Age in years | | | | |
| Mean ± s.d.[b] | 66 ± 14.3 | 60 ± 16 | 59 ± 16 | |
| Median | 66 | 61 | 59 | |
| Range | 29–90 | 17–94 | 21–83 | |
| ≤ 60 | 11 (30.6%) | 31 (44.9%) | 39 (54.2%) | |
| > 60 | 25 (69.4%) | 38 (55.1%) | 33 (45.8%) | 0.066 |
| PS[c] | | | | |
| 0–1 | 27 (75%) | 57 (82.6%) | 58 (80.6%) | |
| 2–4 | 9 (25%) | 8 (11.6%) | 11 (15.3%) | |
| Unknown | 0 | 4 (5.8%) | 3 (4.1%) | 0.255 |
| B-symptoms present | 6 (18.2%) | 21 (30.9%) | 20 (29%) | 0.388 |
| Stage | | | | |
| I–II | 29 (82.9%) | 39 (56.5%) | 46 (63.9%) | |
| III–IV | 7 (17.1%) | 30 (43.5%) | 26 (36.1%) | 0.029 |
| Extranodal sites at presentation | 23 (63.9%) | 24 (34.8%) | 36 (50%) | 0.012 |
| > 1 extranodal site | 2 (5.6%) | 4 (5.8%) | 5 (6.9%) | 0.945 |
| Bulky disease | | | | |
| ≥ 5 cm | 20 (76.9%) | 50 (75.8%) | 56 (78.9%) | 0.909 |
| LDH | | | | |
| ≥ 1.5 normal | 8 (22.2%) | 21 (30.4%) | 19 (30.7%) | |
| < 1.5 normal | 21 (58.3%) | 41 (59.4%) | 46 (63.9%) | 0.783[d] |
| Unknown | 7 (19.5%) | 7 (10.2%) | 7 (5.4%) | |
| Bone marrow involvement | 3 (8.3%) | 12 (17.4%) | 4 (5.6%) | 0.066 |
| IPI risk group[e] | | | | |
| Risk group 1 | 22 (61.1%) | 41 (59.4%) | 41 (56.9%) | |
| Risk group 2–4 | 14 (38.9%) | 28 (40.6%) | 31 (43.1%) | 0.908 |

[a]IB NHL = immunoblastic NHL; CB-Poly NHL = centroblastic polymorphic subtype NHL; CB NHL = centroblastic NHL. [b]s.d. = standard deviation. [c]PS = Performance status according to the WHO criteria. [d]*P*-value reflects to the number of patients of whom the LDH was known. [e]IPI risk groups as defined by Shipp et al (1993).

**Table 2** Place of extranodal sites at initial diagnosis

| | IB NHL[a] | CB-Poly NHL | CB NHL |
|---|---|---|---|
| Total no. of patients | 36 | 69 | 72 |
| No of patients with extranodal sites | 23[b] | 24 | 36 |
| Nose/throat region including sinus | 10 (43.4%)[c] | 8 (33.3%) | 12 (33.3%) |
| Central nervous system | 2 (8.7%) | 3 (12.5%) | 1 (2.7%) |
| Testis | 3 (13%) | 2 (8.4%) | 1 (2.7%) |
| Gastro-intestinal | 3 (13%) | 4 (16.7%) | 5 (13.8%) |
| Cutis/subcutis | 3 (13%) | 3 (12.5%) | 5 (13.8%) |
| Bones (not bone marrow) | 2 (8.6%) | 5 (20.8%) | 5 (13.8%) |
| Thyroid gland | 0 (0%) | 0 (0%) | 3 (8%) |
| Other | 3 (13%) | 3 (12.5%) | 9 (25%) |

[a]IB NHL = immunoblastic NHL;CB-Poly NHL = centroblastic polymorphic subtype NHL; CB = centroblastic NHL. [b]1 patient with an IB NHL had 2 and 1 patient had 3 extranodal sites, 4 patients with CB-Poly NHL had 2 extranodal sites, 5 patients with CB NHL had 2 extranodal sites. [c]The percentages reflect to the total number of patients with extranodal localizations within the histological subtype of large B-cell NHL.

**Table 3** Response to first line treatment

| | IB NHL[a] | CB-Poly NHL | CB NHL |
|---|---|---|---|
| Total no. of patients | 36 | 69 | 72 |
| CHOP-like therapy | 20 (55.6%)[b] | 48 (69.6%) | 58 (80.6%) |
| Dose reductions | | | |
| < 50% | 1 (5%) | 15 (31.3%) | 22 (37.9%) |
| ≥ 50% | 2 (10%) | 2 (4.2%) | 2 (3.4%) |
| Radiotherapy | 21 (58.3%) | 56 (81.2%) | 62 (86.1%) |
| Dose reduction RT | 1 (4.8%) | 4 (7.1%) | 4 (6.5%) |
| CHOP-like therapy with radiotherapy | 7 (19.4%) | 35 (50.7%) | 50 (69.4%) |
| CHOP-like therapy only | 13 (36.1%) | 13 (18.8%) | 8 (11.1%) |
| Radiotherapy only | 14 (38.9%) | 21 (30.4%) | 12 (16.7%) |
| No therapy | 2 (5.6%) | 1 (1.5%) | 1 (1.4%) |
| Response to therapy | | | |
| CR[c] | 27 (75%) | 51 (73.9%) | 55 (76.4%) |
| PR | 2 (5.6%) | 5 (7.2%) | 7 (9.7%) |
| SD | 0 | 0 | 1 (1.4%) |
| PD | 5 (13.9%) | 11 (15.9%) | 8 (11.1%) |
| Not evaluable | 2 (5.6%) | 2 (2.9%) | 1 (1.4%) |

[a]IB NHL = immunoblastic NHL; CB-Poly NHL = centroblastic polymorphic subtype NHL;CB = centroblastic NHL. [b]The percentages reflect to the total number of the subentities. [c]CR = complete remission; PR = partial remission; SD = stable disease; PD = progressive disease according to the WHO criteria.

with immunoblastic NHL), age over 80 years (2 patients with immunoblastic NHL, 2 patients with centroblastic polymorphic subtype NHL, 4 patients with centroblastic NHL).

Four patients did not receive any therapy at all because of rapid deterioration (2 patients with immunoblastic NHL), very old age (1 patient with centroblastic polymorphic NHL, 91-year-old patient) and no evidence of disease after surgical resection (1 patient with centroblastic NHL). The dose reductions and response to the CHOP-like regimens and radiotherapy are summarized in Table 3. The response to treatment was similar in the different histological subgroups.

Dose reductions occurred more frequently in patients with a centroblastic polymorphic or centroblastic NHL than in patients with an immunoblastic NHL.

### Clinical outcome

The median follow-up for all 177 patients was 5 years and 4 months and was similar in all histological subtypes (5 years and 4 months for the 72 patients with centroblastic NHL, 5 years and 3 months for the 69 patients with centroblastic polymorphic subtype NHL and 5 years and 8 months for the 36 patients with immunoblastic NHL).

The clinical outcome (last evaluation in December 1996) is summarized in Table 4. The major cause of death was NHL in all three groups.

Multivariate analysis (including sex, B-symptoms, bulky disease ≥ 5 cm, bone marrow involvement, the international prognostic index) showed only prognostic significance in relation to overall and disease-free survival for the international prognostic index (IPI).

In addition to the IPI, histological subclassification had an independent prognostic significance. With regard to disease-free survival, the IPI risk group 2, 3 and 4 together showed a risk ratio of 2.12 compared with risk group 1 ($P = 0.0001$). Patients with an immunoblastic NHL or centroblastic polymorphic NHL showed in comparison to the patients with a centroblastic NHL a risk ratio of

**Table 4** Clinical outcome

| | IB NHL[a] | CB-Poly NHL | CB NHL |
|---|---|---|---|
| Total no. of patients | 36 | 69 | 72 |
| Alive | 14 (38.9%) | 26 (37.7%) | 41 (56.9%) |
| Lost to follow-up | 2 (5.6%) | 1 (1.4%) | 1 (1.4%) |
| Dead | 20 (55.2%) | 42 (60.9%) | 30 (41.7%) |
| Cause of death | | | |
| NHL | 13 (65%)[b] | 28 (66.7%) | 21 (70%) |
| Toxicity of therapy[c] | 2 (10%) | 3 (7.1%) | 1 (3.3%) |
| Second malignancy | 1 (5%) | 1 (2.4%) | 0 |
| Other | 3 (15%) | 7 (16.7%) | 8 (26.7%) |
| Unknown | 1 (5%) | 3 (7.1%) | 0 |

[a]IB NHL = immunoblastic NHL; CB-Poly NHL = centroblastic polymorphic subtype NHL; CB = centroblastic NHL. [b]Percentages reflect to the number of dead patients per subentity. [c]Toxicity of salvage regimens because of progressive disease, including autologous bone marrow transplantations.

2.01 ($P$ = 0.0105) and 2.02 ($P$ = 0.0019) respectively.

With regard to overall survival, the IPI risk group 2, 3 and 4 together showed a risk ratio of 2.735 ($P$ = 0.0001) in comparison with risk group 1; the immunoblastic or centroblastic polymorphic NHL subtypes showed a risk ratio of 1.819 ($P$ = 0.0391) and 1.712 ($P$ = 0.0248) respectively in comparison with centroblastic NHL.

The overall survival and disease-free survival curves by histologic subtypes are shown in Figure 2. The 5-year disease-free survival for patients with a centroblastic NHL was 53.4%, for patients with an immunoblastic or centroblastic polymorphic subtype NHL 26.9% and 32% (log-rank test stratified by the risk groups as defined by the IPI: $P$ = 0.004); the 5-year overall survival was 56.3%, 39.1% and 41.6% respectively (log-rank test, stratified by the risk groups as defined by the IPI: $P$ = 0.022). The patients with centroblastic NHL thus had a better disease-free and overall survival compared with patients with a centroblastic polymorphic subtype or immunoblastic NHL, even when adjusting for the IPI.

This retrospective study confirms the value of the IPI criteria (Shipp et al, 1993): the 5-year overall survival of patients with 0 or 1 risk factor (risk group 1) was 61.8% vs 26.6% for the patients with 2 or more risk factors (risk groups 2–4; log-rank test, stratified by histological subtype: $P < 0.0001$); the 5-year disease-free survival for the patients with 0 or 1 risk factor (risk group 1) was 51.4% vs 21.4% for the patients with 2 or more risk factors (risk groups 2–4; log-rank test, stratified by histological subtype: $P < 0.0001$).

We investigated the significance of the relative percentage of immunoblasts present in the centroblastic polymorphic subtype. Owing to the small numbers in the groups, the significance of the of the percentage of immunoblasts on overall or disease free survival in these subgroups could not be reliably interpreted.

### DISCUSSION

This retrospective study shows that patients with an immunoblastic and centroblastic polymorphic subtype NHL have a worse prognosis than patients with a diffuse centroblastic NHL, independent of the clinical prognostic criteria as defined by the IPI (Shipp et al, 1993). Although the initial response to therapy was the same for the three histological subtypes, the patients with immunoblastic and centroblastic polymorphic subtype NHL had a higher risk of recurrence of their NHL than the patients with centroblastic NHL, resulting in a significantly worse disease-free and overall survival. The patients with a centroblastic NHL did show a better outcome, despite the fact that dose reductions in their chemotherapy schedule occurred more frequently than in the patients with an immunoblastic NHL and with a similar frequency in the patients with a centroblastic polymorphic NHL (Table 3). Therefore, the dose intensity of treatment cannot explain the different outcome in the three histological subgroups.

Our findings are consistent with the results of the recent study of Engelhard et al (1997) and former publications (Rosenberg et al, 1982; Stein and Dallenbach, 1992). Other authors, however, could not demonstrate a prognostic significance of histological subtyping in large B-cell NHL (Simon et al, 1988; Kwak et al, 1991; Dumont et al, 1992; Koza et al, 1992). The reason for this discrepancy is not clear, but may be due to the relatively small number of patients with immunoblastic NHL and/or a different setting of criteria in most of these retrospective studies. The discrepancy of the outcome of these studies can also be explained by the hypothesis that the immunoblastic, centroblastic polymorphic subtype and centroblastic NHL are not separate disease entities, but may represent the extremes of one biological entity.

As many other studies have done, our study confirms the value of the IPI (Shipp et al, 1993) for patients with NHL of intermediate and high-grade malignancy.

In addition to the prognostic significance of histological subtyping of large B-cell NHL, we analysed whether the different histological categories are related to specific differences in clinical presentation. The patients with immunoblastic NHL tended to be older than the patients with centroblastic or centroblastic polymorphic subtype NHL. This has also been reported by Kwak et al (1991). The patients with an immunoblastic NHL presented more frequently with stage I or II and with one extranodal site than patients with a centroblastic or centroblastic polymorphic subtype of NHL.

None of the subtypes of large B-cell NHL showed preference for localization in a particular site, except localization of centroblastic NHL in the thyroid, but the numbers are too small to draw definite conclusions. We found also no major differences in the sites of recurrent disease for the histological subtypes of large B-cell NHL.

Several studies report a more frequent involvement of central nervous system and bone marrow in patients with an immunoblastic NHL (Koza et al, 1992; Rodriquez and Khan, 1995). This finding has been used to justify central nervous system prophylaxis in the treatment schedules of these patients (Koza et al, 1992). Our findings, however, give no support for this approach. These results are in line with other studies (Simon et al, 1988; Murphy et al, 1989; Hvizdala et al, 1991; Kwak et al, 1991; Dumont et al, 1992; Engelhard et al, 1997; Bos et al, 1998). Therefore, histological subclassification of large B-cell NHL should not be used to select patients for central nervous system prophylaxis.

The reproducibility of the subclassification of the large B cell NHL is a well-known problem. Although the rate of discrepancy between the two contributory haematopathologists was within acceptable limits in this study (6.2% of all cases), additional immunohistochemical stainings that can contribute to improvement of the reproducibility and discrimination between the several subgroups of NHL might be helpful. In our study, immunohistochemical staining with CD138 (Syndecan-1) and VS38c, both

 *© Cancer Research Campaign 1999*

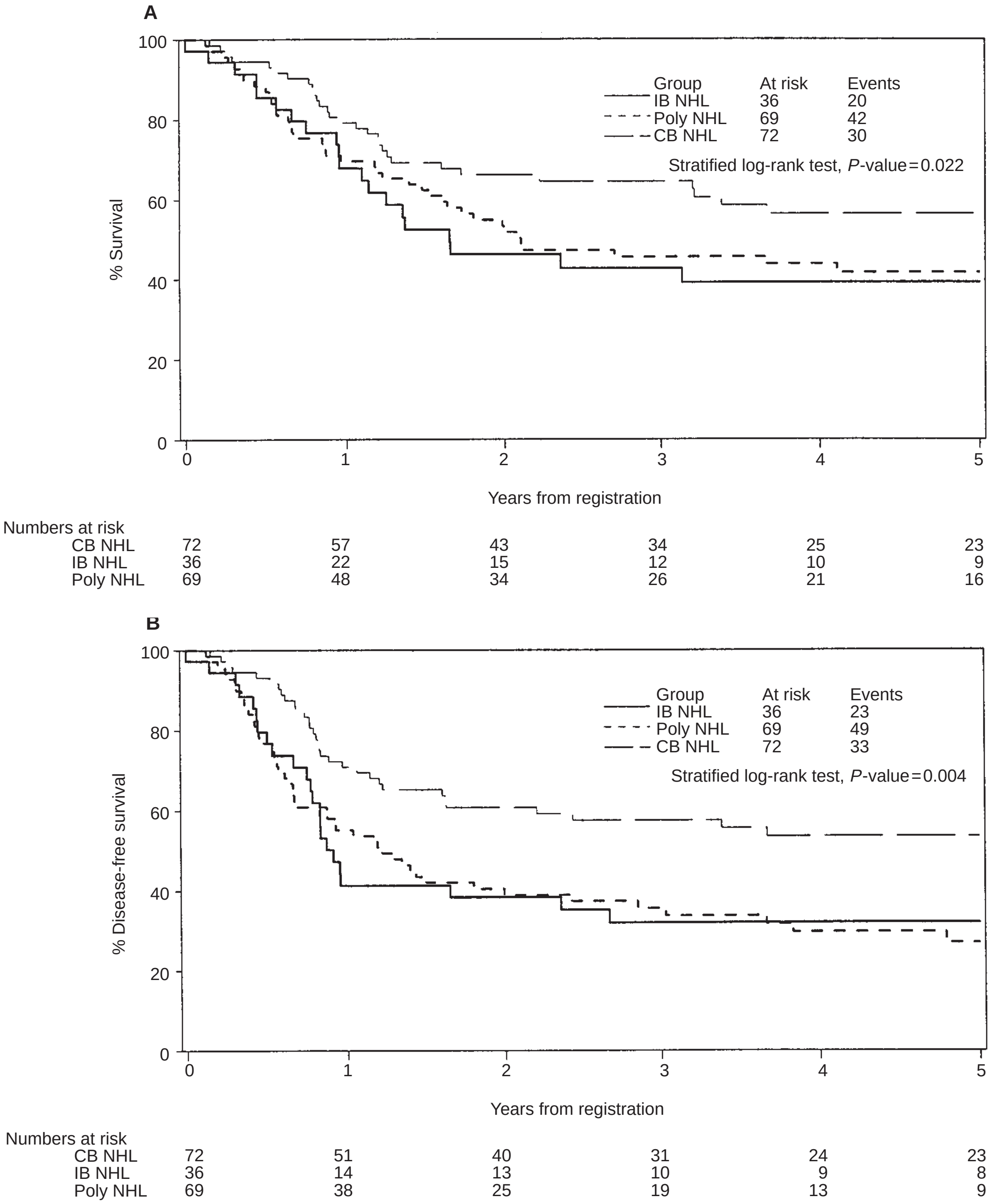

**Figure 2** Overall (**A**) and disease-free (**B**) survival of 177 patients with a diffuse large cell B-cell NHL by histological subentity (log-rank test stratified by IPI criteria). IB NHL = immunoblastic NHL, Poly NHL = centroblastic polymorphic subtype NHL, CB NHL = centroblastic NHL

staining plasma cells and plasmacytoid blasts, was insufficiently reliable to add significantly to the quantification of immunoblasts. This can be partly explained by the fact that this marker was quite sensitive to decline of staining intensity upon storage of archival unstained paraffin sections. In acquired immunodeficiency syndrome related large B-cell NHL CD138 (Syndecan-1) expression was only found in the immunoblastic subtype, although the degree of expression varied considerably from 0 to 75% (Carbone et al, 1998), supporting the view that immunohistochemical staining with CD138 and similar antibodies alone is of minor additional value for subclassification of large B-cell NHL.

The results of the study of Engelhard et al (Engelhard et al, 1997) and our study support the prognostic significance of morphological distinction between immunoblastic, centroblastic polymorphic and centroblastic NHL. This finding merits further exploration in larger prospective studies with uniformly staged patients treated with standardized therapies to judge the value of histological subclassification of large B-cell NHL as a guideline in therapy choice. Immunohistochemical and molecular biological data may prove to be of additional value to distinguish reproducible subentities of diffuse large B-cell NHL and may support the inherently subjective analysis of cellular morphology.

This approach may ultimately help to identify patients who may require other than standard treatment in order to improve their prognosis (Canellos, 1997).

### ACKNOWLEDGEMENTS

We thank our colleagues Klaas J Roozendaal (Onze Lieve Vrouwe Gasthuis, Amsterdam), Ir Koos M. van de Hoeven (Ziekenhuis Amstelveen, Amstelveen) and Marcel Soesan (Slotervaartziekenhuis, Amsterdam) for their kind co-operation in providing us with the clinical data of some of the patients who were included in this study. We are indebted to the members of the CCCA Lymphoma Review Panel between 1984 and 1994: Peter v Heerde, Prof Stefan Pals, Prof Chris JLM Meijer, L Arnold Noorduyn and Prof. Paul vd Valk. We thank Donné Majoor for the expert technical assistance in immunohistochemistry.

### REFERENCES

Berard CW and Hutchison RE (1997) The problem of classifying lymphomas: an orderly prescription for progress. *Ann Oncol* **8** (suppl 2): S3–S9

Bos GJM, van Putten WLJ, v.d. Holt B, v.d. Bent M, Verdonck LF and Hagenbeek A on behalf of the Dutch HOVON group (1998) For which patients with aggressive non-Hodgkin's lymphoma is prophylaxis for central nervous system disease mandatory? *Ann Oncol* **9**: 191–194

Canellos GP (1997) CHOP may have been part of the beginning but certainly not the end: issues in risk-related therapy of large-cell lymphoma. *J Clin Oncol* **15**: 1713–1716

Carbone A, Gaidano G, Gloghini A, Larocca LM, Capello D, Canzonieri V, Antinori A, Tirelli U, Falini B and Dalla-Favera R (1998) Differential expression of BCL-6, CD138/Syndecan-1, and Epstein-Barr Virus-Encoded Latent Membrane Protein-1 identifies distinct histogenetic subsets of acquired immunodeficiency syndrome-related non-Hodgkin's lymphomas. *Blood* **91**: 747–755

Dumont J, Charpy-Validire P, Raphael M, Ulliez M, Le Doussal V, Barge J and Mosseri V (1992) Lymphomas à grandes cellules: influence des groupes histologiques sur le pronostic. Etude de 210 cas soumis aux mêmes protocoles thérapeutiques. *Arch Anat Cytol Path* **40**: 110–114

Engelhard M, Brittinger G, Huhn D, Gerharts HH, Meusers P, Siegert W, Thiel E, Wilmanns W, Aydemir U, Bierwolf S, Griesser H, Tiemann M and Lennert K (1997) Subclassification of diffuse large B-cell lymphoma according to the Kiel classification: distinction of centroblastic and immunoblastic lymphomas is a significant prognostic risk factor. *Blood* **89**: 2291–2297

Harris NL, Jaffe ES, Stein H, Banks PM, Chan JKC, Cleary ML, Delsol G, de Wolf-Peeters C, Falini B, Gatter KC, Grogan TM, Isaacson PG, Knowles DM, Mason DY, Muller-Hermelink HK, Pileri SA, Piris MA, Ralfkiaer and Warnke RA (1994) Revised European–American Classification of Lymphoid Neoplasms: a proposal from the International Lymphoma Study Group. *Blood* **84**: 1361–1392

van Heerde P, Meijer CJLM, Noorduyn LA and van der Valk P (1996) *An Atlas and Textbook of Malignant Lymphomas*. *Cytology, Histopathology and Immunochemistry*, pp 25–62. Harvey Miller Publishers, Manson Publishing: London

Hvizdala EV, Berard C, Callihan T, Falletta J, Sabio H, Shuster JJ, Sullivan M and Wharam MD (1991) Nonlymphoblastic lymphoma in children – histology and stage-related response to therapy: a pediatric oncology group study. *J Clin Oncol* **9**: 1189–1195

Jaffe ES, Harris NL, Chan JKC, Stein H and Vardiman JW (1997) Society for Hematopathology Program. Proposed World Health Organization Classification of neoplastic diseases of hematopoietic and lymphoid tissues. *Am J Surg Pathol* **21**: 114–121

Koza I, Mardiak J, Bohunicky L, Svancárová L, Fuchsberger P, Gyárfás J, Horák I, Spánik S, Sufliarsky J, Thalmeinerová Z and Cerny V (1992) Intensive combination chemotherapy (TTL-I protocol) of large cell and immunoblastic lymphomas – long-term observation. *Neoplasma* **39**: 43–47

Kwak L, Wilson M, Weiss LM, Horning SJ, Warnke RA and Dorfman RF (1991) Clinical significance of morphologic subdivision in diffuse large cell lymphoma. *Cancer* **68**: 1988–1993

Lennert K and Feller AC (1990) *Histopathologie der Non-Hodgkin-Lymphome (nach der aktualisierten Kiel-Klassification*), 2nd ed., pp 100–122. Springer: Berlin

Murphy SB, Fairclough DL, Hutchison RE and Berard CW (1989) Non-Hodgkin's lymphoma of childhood: an analysis of the histology, staging, and response to treatment of 338 cases at a single institution. *J Clin Oncol* **7**: 186–193

Rodriquez JM and Khan AA (1995) Combined chemotherapy and radiotherapy in diffuse large cell immunoblastic lymphoma: a phase II study of CHOP/bleomycin/methotrexate alternating with ifosfamide/methotrexate/etoposide. *Clin Oncol* **7**: 113–116

Rosenberg SA, Berard CW, Brown BW, Burke J, Dorman RF, Glatstein E, Hoppe RT and Simon R Non-Hodgkin's Lymphoma Pathologic Classification Project (1982) National Cancer Institute sponsored study of classifications of non-Hodgkin's Lymphomas. Summary and description of a working formulation for clinical usage. *Cancer* **49**: 2112–2135

Shipp MA, Harrington DP, Anderson JR, Armitage JO, Bonadonna G, Brittinger G, Cabanillas F, Canellos GP, Coiffier B, Connors JM, Cowan RA, Crowther D, Dahlberg S, Engelhard M, Fisher RI, Gisselbrecht C, Horning SJ, Lepage E, Lister TA, Meerwaldt JH, Monterrat E, Nissen NI, Oken MM, Peterson BA, Tondini C, Velasquez WA and Yeap BY (1993) A predictive model for aggressive non-Hodgkin's lymphoma. The International Non-Hodgkin's Lymphoma Prognostic Factors Project. *N Engl J Med* **329**: 987–994

Simon R, Durrleman S, Hoppe RT, Bonadonna G, Bloomfield CD, Rudders RA, Cheson B and Berard CW (1988) The non-Hodgkin Lymphoma Classification Project. Long term follow-up of 1153 patients with non-Hodgkin lymphomas. *Ann Intern Med* **109**: 939–945

Stein H and Dallenbach F (1992) Diffuse large cell lymphomas of B and T cell type. In *Neoplastic Hematopathology*, Knowles DM. (ed) pp 675–714. Williams & Wilkins: Baltimore.

Wijdenes J, Vooijs WC, Clément C, Post J, Morard F, Vita N, Laurent P, Sun R-X, Klein B and Dore J-M (1996) A plasmocyte selective monoclonal antibody (B-B4) recognizes syndecan-1. *Br J Haematol* **94**: 318–323

*© Cancer Research Campaign 1999*
